# Manganese-enhanced MRI for the detection of metastatic potential in colorectal cancer

**DOI:** 10.1186/s41747-017-0024-3

**Published:** 2017-11-02

**Authors:** liang Wen, Xinan Shi, Liping He, Yi Lu, Dan Han

**Affiliations:** 1 Radiology department , The First Affiliated Hospital of Kunming Medical University, 295 Xichang Road, Kunming city, Yunnan province 650000 People’s Republic of China; 2 Kunming Medical University, Kunming City, People’s Republic of China

**Keywords:** Colorectal cancer, Contrast-enhanced magnetic resonance imaging (MRI), Manganese, Metastatic potential, Manganese superoxide dismutase

## Abstract

**Background:**

To study manganese superoxide dismutase (MnSOD) expression, manganese-enhanced magnetic resonance imaging (MEMRI) appearance and its relation to metastatic potential in colorectal cancer (CRC).

**Methods:**

CRC cells SW620, HCT116, LoVo, SW480, DLD-1, HCT15, Caco-2 and their normal counterpart CCD841 CoN were chosen, based on differential aggressiveness, to undergo Western blot analysis for assessment of MnSOD expression, reported as proportion of readings to internal reference (glyceraldehyde-3-phosphate-dehydrogenase). Based on the results of the invasion assay, HCT15, DLD-1, LoVo and SW620 cells and corresponding xenografts underwent MEMRI. The differences of average T1-value shortening were compared.

**Results:**

MnSOD expression in SW620, HCT116, LoVo, SW480, DLD-1, HCT15, Caco-2 and CCD841 CoN cells (0.255 ± 0.018 (mean ± standard deviation), 0.289 ± 0.028, 0.438 ± 0.028, 0.337 ± 0.025, 0.777 ± 0.031, 1.045 ± 0.038, 0.163 ± 0.035 and 0.185 ± 0.038, respectively) was not correlated with Invasion Index (22.6 ± 0.7, 17.0 ± 0.6, 20.9 ± 0.6, 9.7 ± 0.4, 7.5 ± 0.3, 8.3 ± 0.2, 12.6 ± 0.5 and 0) (r = − 0.204, *p* = 0.627). In highly aggressive cells (SW620, LoVo), T1 shortening (289.33 ± 0.57, 268.45 ± 6.87 ms, respectively) was greater than that in lower counterparts (148.68 ± 3.99 ms in DLD-1, 128.60 ± 1.96 in HCT15) (*p* < 0.001). Both 5- and 10-mm group SW620 and/or LoVo tumours showed greater T1 shortening (≥600 ms) than DLD-1 and HCT15 (≤350 ms) (*p* < 0.001, *p* = 0.005, *p* = 0.010).

**Conclusions:**

MEMRI has the potential to noninvasively distinguish different metastatic potential CRCs. However, the MnSOD expression is not correlated to malignant potential in CRC cells.

## Keypoints

- MEMRI detects metastatic potential in CRC
- MnSOD is upregulated in most CRC cells
- MnSOD expression is not correlated with metastatic potential

## Background

Colorectal cancer (CRC) is the third most common cancer and the fourth most common cancer cause of death globally. The stage at diagnosis, frequently with presence of liver metastases, is the most important prognostic factor. Presently, CRC diagnosis and staging mainly rely on colonoscopy and computed tomography (CT) examination [1]. Colonoscopy is able to detect small, primary lesions while CT enables the assessment of lymph node and liver involvement. However, both examinations are not able to suggest the metastatic potential of the primary lesion which is directly associated with prognosis and therapy selection. Magnetic resonance imaging (MRI) has proved to be more accurate in detecting hepatic metastatic lesions of less than 10 mm compared with CT [2]. Associated with the administration of hepatobiliary-specific contrast agents, MRI has been shown to allow an improved sensitivity [3, 4]. Notably, up to 20% of CRC patients have developed remote metastasis at first diagnosis and between 25 and 50% of postoperative patients develop liver metastasis within 3 years after surgery [5–7]. Thus, though there is no current role for MRI-derived biomarkers in predicting metastatic potential of the primary CRC, this perspective deserves to be explored.

Previous researches have documented that manganese superoxide dismutase (MnSOD) expression or activity is upregulated in CRC in comparison with adjacent normal mucosa; moreover, the expression or activity level of this enzyme was not only associated with the depth of invasion, venous plexus involvement and clinical stage [8–12] but also could be an independent prognostic factor for poor 5-year survival of CRC patients [13]. A similar correlation was also reported for breast, lung and bladder neoplasms [14]. However, more recent studies found no correlation between MnSOD expression or activity and clinical stage in CRC [15, 16].

Based on the fact that Mn is bound to SOD as a cofactor for enzymatic activity [17] and that divalent manganese ion (Mn^2+^) has strong paramagnetic effect to alter the longitudinal relaxation of water [18], we tried to employ MnCl_2_ solution as a contrast agent to perform manganese-enhanced MRI (MEMRI) on human CRC cell lines and subcutaneous xenograft tumours. Our aim was to test the MEMRI capability of noninvasively detecting aggressiveness of CRC and, furthermore, to link the MRI appearance of MnSOD expression in CRC cells.

## Methods

### Cell lines

Human CRC cell lines with high metastatic potential (SW620, HCT116, LoVo) and low metastatic potential (SW480, DLD-1, HCT15, Caco-2) as well as normal colon cells CCD841 CoN were purchased from an American-type culture collection (Manassas, VA, USA). The malignant potential of cancer cells has been documented by previous studies [19–24]. These cell lines were cultured in Roswell Park Memorial Institute 1640 (Sigma-Aldrich, St Louis, MO, USA) medium containing 10% foetal bovine serum (Invitrogen, Rockville, MD, USA) at 37 °C in 5% CO_2_ and 95% humidified air.

### Invasion assay

To verify the cell aggressiveness documented by previous studies [19–24] and justify the cell selection for MEMRI invasion, assay was performed by using transwell inserts coated with matrigel basement membrane matrix (BD Biosciences, San Jose, CA, USA) and incubated in humidified 5% CO_2_ at 37 °C for 72 h. The cells on the top membrane and the bottom surface were observed and recorded under the microscope (Carl Zeiss, Canton, China). Five fields were randomly selected and counted as mean value of cell number in each membrane. The percentage of cells that have migrated through the membrane was calculated and was recorded as the Invasion Index. The experiments were repeated twice and the results were averaged.

### Western blot

Anti-SOD2 and glyceraldehyde-3-phosphate dehydrogenase (GAPDH) were purchased from Cell Signaling (Danvers, MA, USA). Cells were lysed with radio-immunoprecipitation assay buffer containing 1 mM phenylmethanesulphonyl fluoride and protease inhibitor cocktail for 30 min at 4 °C. After centrifugation for 15 min at 13,000 rpm, the supernatants were recovered and the protein concentration was measured by bicinchoninic acid Protein Assay Kit (Thermo Fisher Scientific, Waltham, MA, USA). Equal amounts of cell lysates were resolved in 5 × sodium dodecylsulphate and transferred onto nitrocellulose membranes (Sigma-Aldrich, St Louis, MO, USA). After blocking, the membranes were incubated sequentially with the appropriate diluted primary and secondary antibodies. Proteins were detected by the enhanced chemiluminescence detection system (Amersham, Piscataway, NJ, USA). To ensure equal loading of the samples, the membranes were re-probed with a GAPDH antibody (Cell Signaling Technologies, Danvers, MA, USA). The MnSOD expression level was reported as the proportion of readings to internal reference GAPDH.

### In vitro MRI

Based on results of the invasion study, two cells with the highest Invasion Index and the two least aggressive cells were selected to test their Mn uptake at MRI, using normal cell CCD841 CoN as control. MnCl_2_ was dissolved in the culture medium at 0.1 mM for cell lines in the exponential growth phase. After incubation for 60 min, the cells underwent centrifugation at 1000 g for 10 min at 37 °C, 5% CO_2_. The supernatant was removed and fresh medium was added to rinse the cell pellets. After that, the cells were centrifuged and the cell pellets were rinsed again under the same condition for final centrifugation. Corresponding cell lines without administration of MnCl_2_ were also centrifuged in the same way. Then, the cell pellets at a depth of about 10 mm in a Corning tube (1.5 ml) were imaged.

**Fig. 1:**
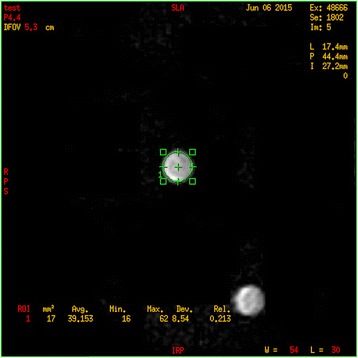
Axial T1-weighted imaging of colorectal cancer (CRC) cells after MnCl_2_ incubation. Regions of interest were manually placed to include the entire cell pellet area

The imaging acquisition was performed using a 1.5-T MRI scanner (Intera, Philips Medical Systems, Best, The Netherlands) equipped with a gradient system with a 30-mT/m maximum amplitude, 0.2-ms minimal rise time and 150-T/m/s maximum slew rate. An eight-element head radiofrequency coil was used. The temperature in the scanner bore was approximately 25 °C. Both T1-weighted imaging (T1WI) and T1-mapping were acquired along axial planes. Firstly, two-dimensional (2D) spin-echo T1WI (2D SE T1WI) was performed with the following technical parameters: time of repetition (TR) 597.22 ms; time of echo (TE) 12.40 ms; field of view (FOV) 60 mm; slice thickness 1 mm; inter-slice interval 1 mm; number of excitations (NEX) 20; matrix 144 × 144. Then, a 2D inversion-recovery turbo spin-echo (2D IR TSE) was performed for T1-mapping, with the following technical parameters: TR 3000 ms; TE 18.5 ms; time of interpulse (TI) 50, 100, 250, 500, 750, 1000, 1250, 1500, 2000 and 2500 ms; slice thickness 1 mm; inter-slice interval 1 mm; FOV 60 mm; NEX 20; matrix 144 × 144. Data calculation and analyses were conducted by a home-made program developed in MatLab (version 7.0; MathWorks, Natick, MA, USA). Both T1 relaxation times with and without Mn administration were calculated on a pixel-by-pixel basis. Two independent measurements were made and the average value was recorded. A region of interest (ROI) of about 17 mm^2^ was manually encircled at the cell-pellet section (Fig. 1). The average T1-value shortening was calculated as follows: T1 value of cells with 0 mM minus T1 value of cells with 0.1 mM. Two independent experiments were carried out.

### Subcutaneous xenografted model on nude mice

This study was approved by the local Animal Care Ethics Committee. Female BALB/c nude mice were kept under specific pathogen-free conditions and cared for in accordance with the guidelines of the Laboratory Animal Ethics Committee. The average weight of the mice was 15 g per mouse. Keeping conditions light/dark the cycle shifted every 12 h. The temperature was 27 °C and the humidity was 50%. The cages, food and water used for the mice were sterilised. For the implanted tumours’ growth assay, two high-metastatic-potential and two low-metastatic-potential cells in 1 × 10^6^/0.2 ml phosphate buffered saline were injected subcutaneously into the right flank of each mouse. Tumour size was measured daily.

### In vivo MRI

At least three nude mice were randomly chosen to undergo MRI when the tumours’ greatest diameter reached 5 and 10 mm, respectively. A 3.0-T scanner (Achieva, Philips Medical Systems, Best, The Netherlands) equipped with a Quasar Dual Gradient system with 80-mT/m maximum amplitude, 0.16-ms minimal rise time and 200-T/m/s maximum slew rate was used. A small-bore, phased-array, radiofrequency coil was used. MnCl_2_ × 4H_2_O (Sigma-Aldrich, St Louis, MO, USA) was dissolved in distilled water to make a 100-mM solution, further diluted with isotonic saline to 50 mM. The mice were anaesthetised with 1.5% isoflurane in the prone position with head down. The temperature of the mice was maintained by an air-warming system at approximately 37 °C. The tumour was placed at the centre of the coil and the MRI acquisitions were performed along the axial plane. Firstly, 2D TSE T1WI was performed with the following parameters: TR 626.17 ms; TE 23 ms; slice thickness 2 mm; inter-slice interval 1 mm; FOV 40 mm; matrix 320 × 320. Secondly, T1- mapping was performed using the variable flip angle method through a three-dimensional, fat-saturated, T1WI turbo field-echo with the following technical parameters: TR 6.9 ms, TE 3.7 ms; flip angle 2°, 10° and 20°; NEX 16; slice thickness 2 mm; inter-slice interval 1 mm; FOV 40 mm; matrix = 256 × 256 [25, 26]. Then, 0.3 μmol/g of 50 mM MnCl_2_ solution was injected intraperitoneally. An intravenous injection of Mn^2+^ was not performed to avoid acute Mn toxicity, taking into consideration the rapid Mn release into the circulation. Manganese-enhanced T1WI and T1-mapping with same parameters were performed again 24 h later. Then, the animals were sacrificed and the excised xenograft tumours were fixed in 10% neutral formalin for routine hematoxylin-eosin staining and CD34 immunohistochemical study. Microvessel density counting was also carried out. The data processing was similar to that previously mentioned. The entire tumour margin on every section was outlined and the average T1 relaxation time was recorded. Measurement for each tumour was repeated three times and average results were recorded.

### Statistical analysis

SPSS version 13.0 (SPSS inc, Chicago, IL, USA) was used to perform the analysis. Because the main purpose of the invasion assay was to verify cell aggressiveness and facilitate cell selection for MEMRI, the statistical analysis was not performed for the results of the invasion assay. The data underwent the Shapiro-Wilk test to analyse their normality. Differences in MnSOD expression level, in vitro Mn uptake of cells and T1-value shortening of xenografts were compared by using one-way analysis of variance, respectively. The Student-Newman-Keuls *q* test was then performed for pairwise comparison. Correlation between MnSOD expression and aggressiveness was analysed by Pearson linear correlation analysis. The results with a *p* value lower than 0.050 were reported as statistically significant.

## Results

### Aggressiveness of cells

**Fig. 2:**
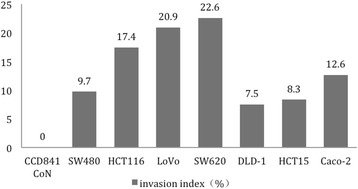
Invasion assay results of cells. SW620, LoVo and DLD-1, HCT15 have shown the highest and lowest Invasion Index, respectively

CRC cells SW620, HCT116, LoVo, SW480, DLD-1, HCT15 and Caco-2 showed various metastatic potentials. SW620 and LoVo had the highest Invasion Index whereas DLD-1 and HCT15 had the lowest one. Normal mucosal cells CCD841 CoN showed an Invasion Index of 0 (Fig. 2).

### MnSOD expression in cell lines

**Fig. 3:**
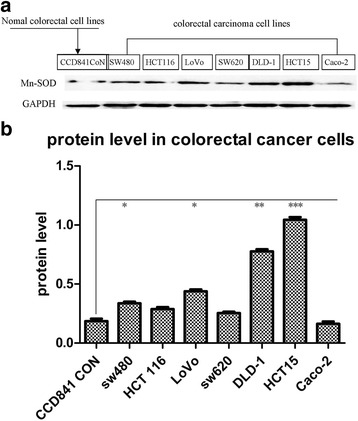
Human colorectal cell cancer and normal cell lines’ manganese superoxide dismutase (MnSOD) expression. **a** Western blot analysis of MnSOD protein with GAPDH as control. **b** Quantitative assessment of MnSOD expression level. Low-metastatic-potential cells DLD-1 and HCT15 have significantly increased expression, high-metastatic-potential cell LoVo and low-metastatic-potential cell SW480 show increased expression. High-metastatic-potential cell SW620 has increased expression but no statistical significance was found. *p = 0.031, *p* = 0.012; **p = 0.001; ***p < 0.001

All seven CRC cell lines and normal cells expressed certain levels of MnSOD. Cancer cell lines showed higher levels of expression than normal cell lines except for Caco-2 (0.163 ± 0.035), its expression being slightly lower than that of normal cell lines (0.185 ± 0.038), without statistical significance. The cells with the highest expression level of MnSOD were HCT15 and DLD-1 (1.045 ± 0.038, 0.777 ± 0.031), both having a low metastatic potential. Cells with relatively high MnSOD expression encompassed both high- and low-metastatic-potential cells, LoVo, SW620, HCT116 and SW480 (0.438 ± 0.028, 0.255 ± 0.018, 0.289 ± 0.028 and 0.337 ± 0.025, respectively). Compared with their normal counterpart, DLD-1 and HCT15 cells demonstrated a marked increase of MnSOD expression (*p* = 0.001, *p* < 0.001) while the MnSOD expression of SW480 and LoVo cells was increased (*p* = 0.031, *p* = 0.012). Although SW620 and HCT116 showed a higher MnSOD expression than normal cells, no statistical significance was found (*p* = 0.067) (Fig. 3). Regression analyses did not find a significant correlation between the expression level of MnSOD and cell aggressiveness (r = − 0.204, *p* = 0.627) (Table 1).

**Table 1:** Manganese superoxide dismutase (MnSOD) expression level and Invasion Index of cells

	CCD841 CoN	DLD-1	HCT15	SW480	Caco-2	HCT116	LoVo	SW620
MnSOD expression level	0.185 ± 0.038	0.777 ± 0.031	1.045 ± 0.038	0.337 ± 0.025	0.163 ± 0.035	0.289 ± 0.028	0.438 ± 0.028	0.255 ± 0.018
Invasion Index (%)	0	7.5 ± 0.3	8.3 ± 0.2	9.7 ± 0.4	12.0 ± 0.5	17.0 ± 0.6	20.0 ± 0.6	22.0 ± 0.7

MnSOD data are mean and standard deviation of the proportion of readings to internal reference (glyceraldehyde-3-phosphate-dehydrogenase)

### In vitro MEMRI of Mn uptake in cell lines

**Fig. 4:**
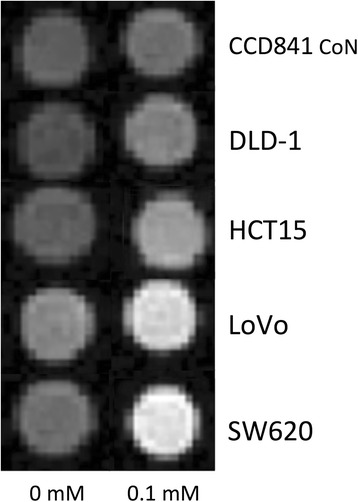
In vitro manganese-enhanced magnetic resonance imaging of cell lines. T1-weighted imaging of cell pellets incubated with and without 0.1-mM MnCl_2_. Cancer cells enhance more markedly than do normal cells and high-metastatic-potential cells SW620 and LoVo enhance the most

All five cell lines incubated in the medium with 0.1-mM MnCl_2_ exhibited a certain degree of Mn uptake shown on T1WI as increased signal intensity compared with cells without the administration of MnCl_2_ (Fig. 4). Further T1-mapping analysis demonstrated that the average T1-value shortening in SW620 and LoVo cells (289.33 ± 0.57 and 268.45 ± 6.87 ms) was significantly higher than DLD-1 and HCT15 (148.68 ± 3.99 and 128.60 ± 1.96 ms) (*p* < 0.001 for both). All cancer cells have shown significantly greater T1-value shortening compared with normal cell (*p* < 0.001) (Table 2).

**Table 2:** Comparison of T1-value shortening in cells at manganese-enhanced magnetic resonance imaging

Cell lines	‾Mean ± standard deviation	F	*P*
SW620	289.33 ± 0.57	2039.259	<0.001
LoVo	268.45 ± 6.87		
HCT15	128.60 ± 1.96		
DLD-1	148.68 ± 3.99		
CCD841 CoN	65.12 ± 0.12		

High-metastatic-potential cells SW620 and LoVo show significantly greater T1 shortening than low-metastatic-potential cells DLD-1 and HCT15 (*p* < 0.001, analysis of variance). Measurement is based on two independent experiments

### Subcutaneous tumour MRI and histopathology

**Fig. 5:**
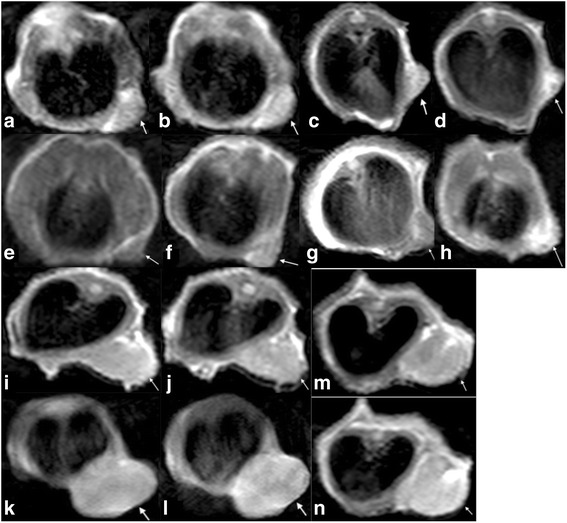
T1-weighted imaging of pre- and post-Mn administration in 5- and 10-mm group xenografts. Arrows in the images from **a** to **n** point to subcutaneous tumour. Images **a**, **c**, **e**, **g**, **i**, **k**, **m** and **b**, **d**, **f**, **h**, **j**, **l** and **n** represent pre- and post-Mn administration, respectively. Images **a**, **b** and **k**, **l** are low-metastatic-potential DLD-1 tumours. Images **c** and **d** are of a high-metastatic-potential SW620 tumour. Images **e**, **f** and **i**, **j** are low-metastatic-potential HCT15 tumours. Images **g**, **h** and **m**, **n** are high-metastatic-potential LoVo tumours. SW620 and LoVo tumours display greater enhancement than DLD-1 and HCT15 tumours

**Fig. 6:**
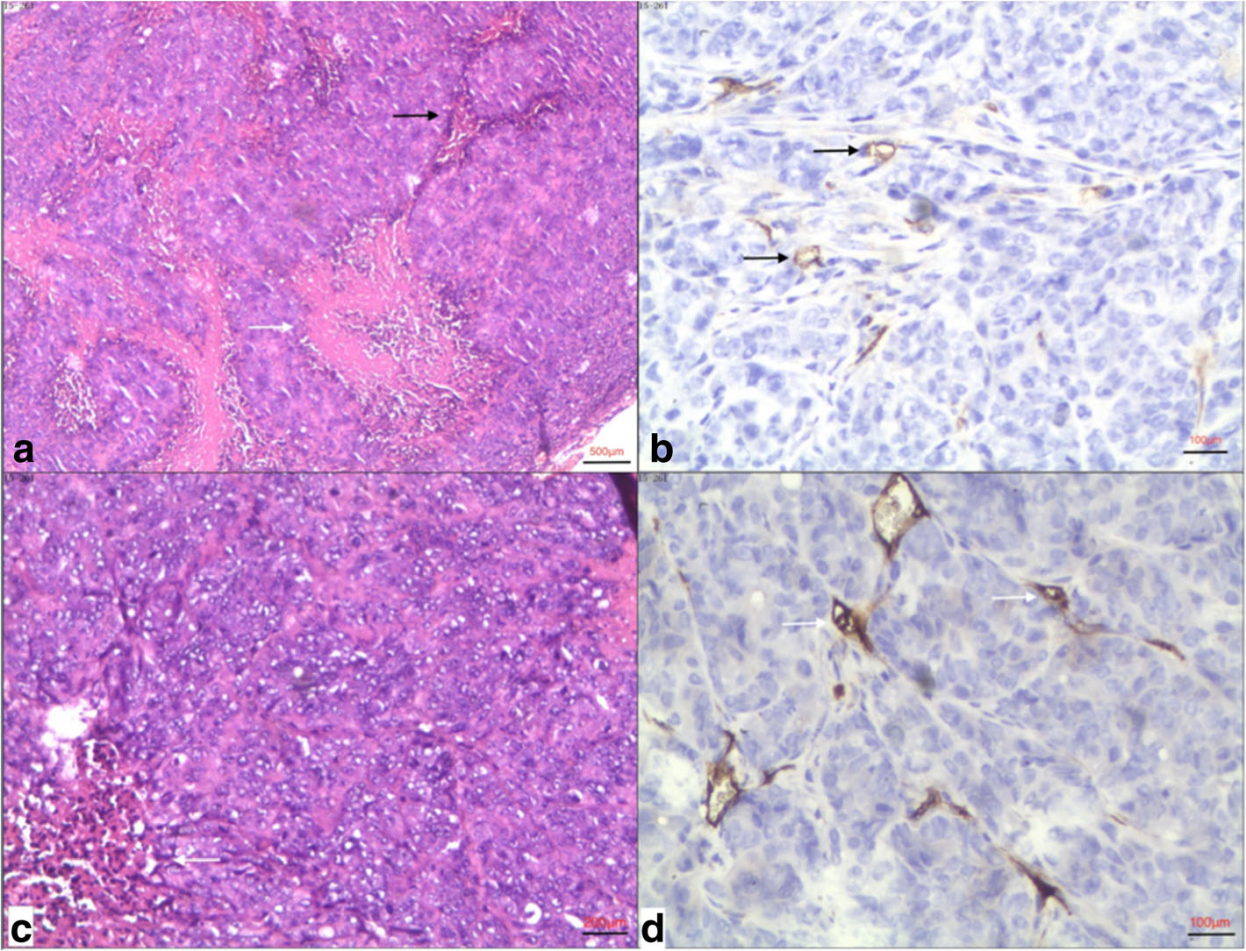
**a** and **b** are a histological photomicrographs (hematoxylin and eosin × 40) and CD34 immunohistochemical staining (×200) of a DLD-1 tumour, respectively. In **a**, packed tumour cells with little interstitium (black arrow) and discrete necrosis (white arrow) are shown. In **b**, black arrows point to sparse microvessels. **c** and **d** are a histological photomicrograph (haematoxylin and eosin × 100) and CD34 immunohistochemical staining (×200) of a SW620 tumour, respectively. In **c**, relatively little interstitium (white arrow) is noted in packed tumour cells. In **d**, white arrows indicate sparse microvessels

The numbers of successfully developed xenografts is shown in Table 3. Twenty-nine tumours displayed iso- or slightly higher signal intensity relative to muscle on 2D TSE T1WI. On T1 mapping, the difference in average tumour T1-value shortening in the 5-mm group compared by one-way analysis of variance showed that both SW620 (*n* = 5; 730.56 ± 33.48 ms) and LoVo xenografts (*n* = 3; 637.57 ± 47.62 ms) were significantly greater than DLD-1 (*n* = 5; 265.28 ± 19.59 ms) and HCT15 (*n* = 4; 262.60 ± 46.48 ms) (*p* < 0.001 for both). One-way analysis of variance in the 10-mm group tumours has shown that T1 shortening in LoVo xenografts (*n* = 3; 620.33 ± 88.60 ms) was significantly greater than in DLD-1 (*n* = 6 ; 346.82 ± 108.04 ms) and HCT15 (*n* = 3; 341.97 ± 112.12 ms) (*p* = 0.005, *p* = 0.010, respectively). However, no statistical difference was found between LoVo and SW620, as well as DLD-1 and HCT15 tumours in neither the 5- nor the 10-mm group (*p* = 0.107) (Table 4, Fig. 5). The routine hematoxylin-eosin staining verified that all 29 xenografts were moderate- to low-differentiated adenocarcinoma. Under the light microscope the packed neoplastic cells were patchily distributed with sparse interstitium and discrete coagulative necrosis was found. Sparse microvessels were shown on CD34 staining, the minimum and maximum microvessel density counts were 11.4 and 20, respectively, with an average count of 15.6 per × 200 field (Fig. 6).

**Table 3:** Xenografts successfully developed in 5- and 10-mm size groups

	Tumour number	
Tumour	5 mm	10 mm
LoVo	3	3
SW620	5	0
DLD-1	5	6
HCT15	4	3

**Table 4:** T1-value shortening of xenografts in 5- and 10-mm size groups

	Greatest diameter of tumours (mm)	
Tumours	5 mm	10 mm
SW620	730.56 ± 33.48^c,d^	—
LoVo	637.57 ± 47.62^c,d^	620.33 ± 88.60^c,d^
DLD-1	265.28 ± 19.5^a,b^	346.82 ± 108.04^b^
HCT15	262.60 ± 46.48^a,b^	341.97 ± 112.12^b^

Data are expressed as mean ± standard deviation

Symbols indicate that the difference between SW620 (^a^), LoVo (^b^), DLD-1 (^c^) and HCT15 (^d^) is statistically significant

## Discussion

The current approaches for diagnosing and staging CRC are not able to noninvasively define tumour malignant potential that is directly correlated with therapy selection and prognosis. The controversial results regarding the relation between MnSOD expression and tumour malignant potential in CRC reported in clinical researches, and some results concerning MEMRI of neoplasms from recent studies [27–38], inspired us to explore the application of MEMRI in noninvasive evaluation of CRC metastatic potential. In addition, the attempt to correlate MEMRI appearance with MnSOD expression in CRC cells was made.

Our invasion assay has verified the differential metastatic potential of seven CRC cells, which was consistent with the results reported in previous studies. These researches documented that SW620 and LoVo cells were derived from metastatic lymph nodes of colorectal cancer, the tumourigenicity, growth kinetics and metastatic potential of these cells were proved to be more aggressive than in SW480, DLD-1, HCT15 and Caco-2 that were derived from primary tumours [19–24]. Our study confirmed that SW620, LoVo and DLD-1, HCT15 have shown the highest and lowest Invasion Index, respectively.

As one of the most important antioxidant enzymes, MnSOD protein has been considered downregulated in malignant neoplasms in the earliest studies, though subsequent researchers have found increased MnSOD expression in malignant mesothelioma and some neoplasms arising from thyroid, kidney and central nervous system cells as well as in CRC in most clinical studies [14]. This was verified in our in vitro experiment. However, the relation between MnSOD expression and malignant potential is more complicated. Some clinical studies have shown that MnSOD expression is positively correlated with CRC malignant potential [8–12]. However, our in vitro study found that the cells with the highest MnSOD expression had low metastatic potential, whereas cells with a relatively low expression encompassed both high and low metastatic potentials. This indicates that the expression of MnSOD in CRC cells was not correlated to malignant potential. Because the expression of MnSOD is a response to oxidative stress [39, 40], the microenvironmental redox status of tumours influences its expression. The different redox status in human body and cell culture environments might contribute to the discrepancy between the present study and previous clinical researches.

In this study, both MEMRI studies in cells and xenografts have shown significant difference in T1 shortening between CRCs with different metastatic potential: highly aggressive cells/tumours showed two to three times greater T1 shortening than less aggressive ones, with high statistical significance. Similar findings were reported by Nofiele et al. in human breast cancer cells [36]. The author observed that the Mn induced a longitudinal relaxation rate (R_1_) in the most aggressive cells significantly higher (two times) than that in less aggressive ones. Based on these results, it could be postulated that MEMRI might be used to distinguish aggressiveness in cancers.

Mn^2^ + is a well-known intracellular contrast agent for MRI. It is taken up and accumulated by cells and shortens the T1 relaxation time of adjacent water thanks to its strong paramagnetic effect. Compared with extracellular agents (such as Gd-based contrast agents), its enhancement effect is more dependent on cell density than on tumour vascularity. At a biochemical level, Mn is involved in mitochondrial function, and the greater the mitochondrial density, the higher the level of Mn uptake. Hence, it could be speculated that tumours with higher cell density and/or mitochondria would enhance more markedly on MEMRI. This was reflected in our study by the fact that the xenografts that were significantly enhanced with Mn administration have invariably shown histological features of packed tumour cells though only sparse microvessels were found.

At molecular level, several factors involving Mn^2+^ uptake in cells have been reported such as voltage-gated Ca^2+^-channel activity, anti-*N*-methyl-D-aspartate, astroglia and microglia activity/cellularity, and metal transporters [41]. However, the mechanism involved in Mn^2+^ uptake in tumour cells is not fully understood. In a study of human breast cancer the overexpression of calcium-sensing receptor (CaSR) in tumour was suggested to be involved in better Mn-enhancement [29]. However, another study attempting to link CaSR expression to Mn-enhancement in human breast cancer cells did not find this correlation [36]. Nevertheless, the authors found that the most aggressive cells that showed the lowest level of CaSR expression enhanced the most markedly whereas the less aggressive cells with higher levels of CaSR expression enhanced only minimally; furthermore, the higher the concentration of MnCl_2_ administration, the greater the enhancement of both cells. This indicates that, although CaSR plays a role in Mn uptake in tumour cells via its ion-channel regulation, the T1 shortening effect of Mn^2+^ on MRI could not be explained solely by ion-channel activity.

Obviously, differential biological behavior of tumours and Mn^2+^ concentration are also involved in Mn-enhancement. The fact that our study employed identical Mn^2+^ concentration for different cells and tumours either in in-vitro or in in-vivo studies, and that different aggressive malignancies still showed differential Mn-enhancement, further underscores the important role of neoplastic cell biological properties in MEMRI appearance. Actually, two researches have documented the relation between the biological properties of tumour cells and the Mn-enhancement effect in MRI. Braun et al. [30] found that some tumour cells with a higher proliferation rate demonstrated more Mn uptake and greater T1 shortening. Saito et al. [41] detected early cell alteration that was identified by cell-cycle and proliferation changes after irradiation exposure both in vitro and in vivo by using MEMRI. Therefore, it seems reasonable to attribute the greater Mn-enhancement in more aggressive CRC to a higher cell proliferation rate.

Apart from Mn uptake, the subcellular location of intracellular Mn^2+^ may also contribute to the enhancement effect. Free Mn^2+^ in cytosol has greater access to water to induce more T1 shortening compared with mitochondrial Mn^2+^. Because of the more severe functional impairment of mitochondria in more malignant tumour cells, the Mn^2+^ entering the mitochondria might be less than that in less aggressive tumour cells, resulting in more Mn^2+^ accumulating freely in cytosol, thus causing greater T1 shortening. Finally, the contribution from Mn^2+^ in the extracellular compartment should probably also not be overlooked [42].

In addition, studies in malignant mesothelioma [34, 43] favoured the hypothesis that greater Mn-enhancement is related to higher MnSOD expression in tumours. However, in our study the relatively low MnSOD expression cells has clearly shown greater Mn-enhancement than in cells with higher expression. Given that MnSOD is located in the mitochondria and needs binding Mn^2+^ to activate it, it was speculated that cells with a lower MnSOD expression have a lower intracellular Mn^2+^ concentration, which leaves more space for further Mn^2+^ uptake. As a consequence, its T1 shortening was greater than in cells with high MnSOD expression. The contraindicated observations indicate that this relation is not consistent in different cancer types.

In summary, the tumour-enhancement effect of Mn appears to be influenced mainly by the following factors: tumour cellularity, ion-channel regulation, cell-cycle and proliferation rate, tumour aggressiveness, subcellular location of Mn^2+^ as well as MRI technical issues such as agent concentration. However, to what extent each single factor contributes to MEMRI appearance under certain imaging condition still needs studies to clarify.

Manganese toxicity is the main obstacle to translate this MEMRI study into clinical application. Finding the appropriate dosage that generates a sufficient enhancement effect with minimum adverse effects would be the prerequisite to translate animal study into clinical use. However, even the most widely used gadolinium-based MRI contrast agents are not free of adverse effects. Recently, they have been found to induce nephrogenic systemic fibrosis in the presence of renal failure [44] and the problem of gadolinium deposition in various organs is of increasing concern [45, 46]. On the other hand, an orally administered Mn agent, such as CMC-001, has shown promising results in terms of reducing toxic effects and improving the detection of liver metastases in both in-vitro studies and clinical trials [3, 47]. Newly developed Mn-based magnetic nanoparticles also have the potential to reduce toxicity and increase the specificity to target tumours [47]. Given that a significant portion of CRC patients developed remote metastasis, either at first diagnosis or after surgery [5–7], MEMRI approaches evaluating tumour metastatic potential prior to the occurrence of remote metastatic disease could provide valuable information for prognosis and therapy planning. This technique might be also applied to other neoplasms other than CRC.

In conclusion, MEMRI showed the potential to noninvasively distinguish high- from low-metastatic-potential CRC by revealing a greater T1 shortening in the former. However, the MnSOD expression in CRC cells did not correlate with malignant potential.

## Abbreviations

2D SE: Two-dimensional spin-echo
2D: Two-dimensional
CaSR: Calcium-sensing receptor
CRC: Colorectal cancer
CT: Computed tomography
FOV: Field of view
GAPDH: Glyceraldehyde-3-phosphate dehydrogenase
MEMRI: Manganese-enhanced magnetic resonance imaging
MnSOD: Manganese superoxide dismutase
NEX: Number of excitations
T1WI: T1-weighted imaging
TE: Time of echo
TR: Time of repetition
